# The Diversity and Floristic Analysis of Rust Diseases in the Sanjiangyuan Forest Plants

**DOI:** 10.3390/jof10060425

**Published:** 2024-06-16

**Authors:** Qi Xu, Luchao Bai

**Affiliations:** 1 College of Agriculture and Animal Husbandry, Qinghai University, Xining 810016, China; xqq1963500437@outlook.com; 2 State Key Laboratory of Plateau Ecology and Agriculture, Qinghai University, Xining 810016, China

**Keywords:** Sanjiangyuan region, rust fungus, taxonomy, diversity, fungal flora

## Abstract

Between 2020 and 2023, rust fungus specimens were collected from the primary forested regions of the Sanjiangyuan area in Qinghai Province, resulting in over 300 samples. A taxonomic and phylogenetic study of the rust fungi from these forests was conducted using morphological and molecular biological techniques. The investigation identified rust fungi from 7 families, 12 genera, 56 species and varieties, including 10 new host records, 1 new record for China, and 2 novel species. The host plants involved belonged to 26 families, 48 genera, and 78 species. Pucciniaceae and Coleosporiaceae were the dominant families, with the genera *Puccinia*, *Melampsora,* and *Gymnosporangium* being prevalent. The rust fungi in the Sanjiangyuan forests showed a biogeographical affinity with the North Temperate Zone. Floristic comparisons revealed a higher similarity with rust fungi from Inner Mongolia, Gansu, and Tibet and a lower similarity with those from Hainan. An analysis of the life forms of rust fungus host plants indicated that herbaceous plants were the most common, followed by shrubs and trees. In different regions of Sanjiangyuan, rust fungi were found as follows: Golog Prefecture with 6 families, 9 genera, and 28 species; Yushu Prefecture with 5 families, 8 genera, and 31 species; Huangnan Prefecture with 5 families, 9 genera, and 26 species; and Hainan Prefecture with 4 families, 5 genera, and 10 species. The families Pucciniaceae, Melampsoraceae, and Coleosporiaceae were common across all four regions. Moreover, the families Rosaceae, Asteraceae, Ranunculaceae, Salicaceae, and Caprifoliaceae were shared among the host plants in these regions.

## 1. Introduction

Rust fungi (Pucciniales) belong to the phylum Basidiomycota, class Pucciniomycetes, and order Pucciniales [1]. To date, there are 14 families, 166 genera, and over 7000 species of rust fungi recorded worldwide [2], with a broad distribution and a wide range of hosts, posing significant threats as pathogens to many plants. Infected plants often display noticeable symptoms such as deformities, clustering, overgrowth, or enlargement [3]. Rust diseases severely impact the growth and development of dominant tree species within forests and understory vegetation, and can even destroy young plantations, reducing the biomass and seed yields of trees and economic crops, thereby seriously impacting the ecological functions of forestry systems, as exemplified by pine gall rust [4], mulberry rust [5], and poplar leaf rust [6]. On the other hand, rust fungi play an essential role in forest ecosystems as living decomposers, crucial for maintaining the material cycle and ecological balance of forests [7]. Historically, numerous domestic scholars have published regional rust fungi checklists or treatises, covering areas including Jilin [8,9,10], Tibet [11,12], Fujian [13,14], Hubei [15,16], the Qinling Mountains [17,18,19], Xinjiang [20,21,22,23], Gansu [24], and Inner Mongolia [25], among others. These publications meticulously list the rust fungi species and systematically analyze the rust fungi of those regions. The present study conducted a survey and sampling of rust fungi in the main forest regions of the Sanjiangyuan area in Qinghai Province, employing both morphological and molecular systematic methods to classify the collected rust fungi, determine the regional rust fungi characteristics, and compile a checklist of rust fungi in the main forest regions of Sanjiangyuan. This research provides a foundation for further studies on rust fungi taxonomy and offers a scientific basis for the prevention and control of rust diseases in the main forest regions of the Sanjiangyuan area.

## 2. Materials and Methods

### 2.1. Sample Collection

The primary forest region of the Sanjiangyuan area is situated in the hinterland of the Qinghai-Tibet Plateau, in the southern part of Qinghai Province, between the geographical coordinates of 89°24′ E to 102°23′ E longitude and 31°39′ N to 36°16′ N latitude. The Sanjiangyuan region’s altitude ranges from 3836 to 6500 m [26]. The annual mean temperature is between −5.6 °C and 3.8 °C, with most areas experiencing an annual mean temperature below 0 °C, decreasing from southeast to northwest. The highest and lowest temperatures occur in July and January, respectively. Precipitation is primarily concentrated between June and September, accounting for approximately 80% of the annual total. The annual average precipitation ranges from 262.2 to 772.8 mm [27,28]. Field investigations were carried out and specimens were collected from the main forest areas of the Sanjiangyuan region from 2020 to 2023. Plant specimens infected by rust fungi should be collected during the growing season of the plant leaves, typically from April to October each year. The collection date, location, altitude, and host information were recorded. Specimens are stored at the Plant Pathology Laboratory of the College of Agriculture and Animal Husbandry, Qinghai University. Collect the number of specimens as shown in Appendix A.

**Figure 1:**
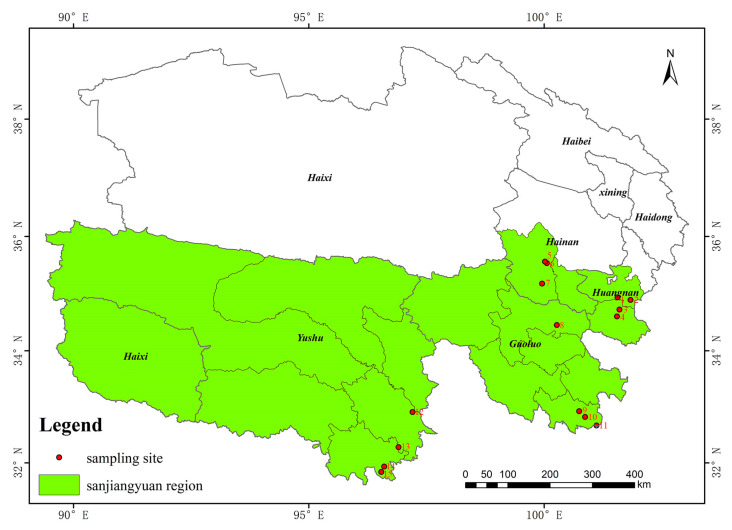
Sampling point distribution map. (1: Lanci; 2: Shuangpengxi; 3: Xibosha; 4: Maixiu; 5: Jiangla; 6: Dongshan; 7: Xihe; 8: Yangyu; 9: Duoke; 10: Makehe; 11: Friendship; 12: Leba; 13: Dongzhong; 14: Jiangxi; 15: Baizha).

The main forest areas of Sanjiangyuan include Maixiu Forest Area, Xibosha Forestry Farm, Shuangpengxi Forestry Farm, Lanci Forestry Farm, Makehe River Forest Area, Yangyu Forest Area, Duoke River Forestry Farm, Friendship Bridge Forestry Farm, Dongzhong Forest Area, Jiangxi Forestry Farm, Leba Forestry Farm, Baizha Forestry Farm, Dongshan Forestry Farm, Xihe Forestry Farm, and Jiangla Forestry Farm. The distribution of sampling sites is illustrated in Figure 1.

### 2.2. Research Methods

#### 2.2.1. Morphological Study

Morphological classification follows the systems in “Fungi of China”, “Manual of Fungal Identification”, and “Dictionary of Fungi”.

(1) Symptom Observation: Using a stereo microscope (Nikon, Tokyo, Japan) to observe the type, morphological characteristics, location on the host plant, color, shape, and distribution of spore heaps (spore structures). Measurements and photographs of the spore heaps (spore structures) are taken.

(2) Spore Structures Observation: Longitudinally sectioned spore heaps (spore structures) are observed for internal structure using an optical microscope (Olympus, Tokyo, Japan).

(3) Spore Characteristics Observation: Mature spore heaps (spore structures) are selected, and spores are randomly picked to observe morphological characteristics using an optical microscope.

(4) Spore Electron Microscopy (Hitachi, Tokyo, Japan) Observation: Conductive adhesive is placed on the sample stage of the scanning electron microscope. Leaves containing spore bodies are placed on the stage and coated with gold using a sputter coater. Surface structure and ornamentation of the spores are observed using a field emission scanning electron microscope, with photographs and records taken.

#### 2.2.2. Molecular Phylogenetic Study

DNA extraction is performed using a modified CTAB method [29]. Amplification of gene sequences for rust fungi ITS and LSU fragments is performed, specifically using primers ITS1F (5′-CTTGGTCATTTAGAGGAAGTAA-3′), ITS4 (5′-TCCTCCGCTTATTGATATGC-3′), NL1 (5′-GCATATCAATAAGCGGAGGAAAAG-3′), and NL4 (5′-GGTCCGTGTTTCAAGACGG-3′). Qualified sequencing sequences are submitted to GenBank to obtain accession numbers. An ML phylogenetic tree is constructed with support rates obtained by bootstrapping (BT) repeated 1000 times, and the phylogenetic relationships between sequences are analyzed.

#### 2.2.3. Floristic Analysis

The floristic geographical characteristics mainly followed the principles of plant floristic division as described by Wu Zhengyi (2003) [30]. The analysis was conducted from the following aspects:

(1) Composition of rust fungi in the main forest areas of Sanjiangyuan

The genera and species of rust fungi and their host plants were enumerated to analyze the proportion of each genus, thereby determining the dominant families and genera. The calculation method was based on the formula proposed by Dong Xueyun et al. [31]:

$$F_{a}=F_{c}>S_{t}/F_{t};\;G_{a}=G_{c}>S_{t}/G_{t}$$

Above formula: *F*_a_ represents the dominant family, *F*_c_ represents the number of species in a family, *S*_t_ is the total number of species, *F*_t_ is the total number of families, *G*_a_ represents the dominant genus, *G*_c_ represents the number of species in a genus, and *G*_t_ is the total number of genera.

(2) Geographic Component Analysis

The geographic distribution of host plant genera and species was examined to clarify the geographical characteristics of the rust fungi flora in the main forest areas of Sanjiangyuan. The known species of rust fungi in the area were listed and compared with those in adjacent regions to calculate their similarity.

(3) Analysis of Host Plant Life Forms: Within the host plants, categorization was made according to their ecological types into trees, shrubs, and grasses. The ecological types of host plants were investigated and analyzed.
(4) Diversity Analysis of Rust Fungi in Different Research Areas: The study areas were divided into four regions based on provincial divisions and forest distribution in the Sanjiangyuan area: Golog Prefecture, Yushu Prefecture, Huangnan Prefecture, and Hainan Prefecture.

① Diversity Calculation of Rust Fungi: The species, number, and frequency of rust fungi in each area were recorded, and the diversity index of rust fungi in different regions was calculated. When assessing species richness, indices such as weighted average number of species, Shannon-Wiener diversity index (H′), and richness index (E) were calculated to analyze the relationship between rust fungi diversity in different regions [32], with the following formulas:

Shannon-Wiener diversity index: $H^{\prime }=-\sum P_{i}\times \mathrm{In}P_{i}$;
Maximum diversity index: ${H^{\prime }}_{\max}=\ln S$;
Evenness index: $E=H^{\prime }/{H^{\prime }}_{\max}$;

Above formula: $P_{i}=n/N$, is the proportion of the ith species, n is the number of individuals of the ith species; N is the total number of all species; S is the number of species.

② Similarity Determination of Rust Fungi: The similarity between different regions was determined by qualitative or quantitative comparisons of species presence, which reflect their relationship and identify the environmental factors or combinations of factors that influence this relationship [32], with the following formula:

$$\mathrm{Sørensen}\;\mathrm{similarity}\;\mathrm{coefficient}:CC_{s}=\frac{2C}{S_{1}+S_{2}}$$

Above formula: *S*_1_ and *S*_2_ are the number of species in community 1 and community 2, respectively; *C* represents the number of common species between communities 1 and 2.

## 3. Results and Analysis

### 3.1. Identification of Rust Fungi Species in the Main Forest Area of Sanjiang Source

Following years of continuous fixed-point surveys and collections in the major forest regions of the Sanjiangyuan area, over 300 rust fungus specimens were collected. A total of 7 families, 12 genera, 56 species, and varieties of rust fungi were identified within these regions, including 1 new record for China and 2 proposed new species, involving 26 families, 48 genera, and 78 species of host plants, with 10 plant species being new records as hosts for rust fungi (Table 1). In this study, two new rust fungi were discovered, parasitic on *Ligularia przewalskii* and *Rheum pumilum*, respectively. The morphological characteristics of the parasitic rust spores on *L. przewalskii* were compared with those of known species, revealing certain differences from other rust spores. Molecular systematics studies of the rust fungi were conducted using molecular biology techniques, showing their affinity with rust fungi of the genus *Puccinia* (GenBank accession number PP469520). Considering that only one species of rust fungus, *P. ligulicola*, has been reported on the host plant *L. przewalskii*, and after consulting relevant literature, we believe that this species is a new one awaiting publication. Similarly, the rust fungus parasitic on *R. pumilum* was identified as *Puccinia* sp. (GenBank accession number PP469561), pending publication. Based on the ITS and LSU segments, an ML system was constructed to build phylogenetic trees, both of which divided the rust fungi in the Three Rivers Source main forest area into seven families, consistent with morphological identification results. The Pucciniaceae family diverges significantly, with genera *Puccinia* and *Uromyces* clustering together in a major branch, while *Gymnosporangium* is dispersed in another well-supported branch. Genera *Ochropsora* and *Nyssopsora* are incorporated into *Gymnosporangium*. Additionally, *Hyalopsora*, *Melampsora*, *Coleosporium*, *Chrysomyxa*, and *Uredo* are grouped into another major branch.

### 3.2. Phylogenetic Analysis

#### 3.2.1. Rust Fungi Composition in the Sanjiangyuan RegionIn

In the primary forest areas of the Sanjiangyuan region, the dominant family of rust fungi is Pucciniaceae, accounting for 36.36% of the total number of rust genera, followed by *Coleosporiaceae*, representing 18.18% of the genera. The prevalent genera are *Puccinia*, constituting 50% of the total species count; *Melampsora*, comprising 12% of the species; and *Gymnosporangium*, making up 11% of the species (Table 2). The host plants’ dominant families are Rosaceae, which represent 16% of the total host species; Asteraceae, accounting for 9%; Ranunculaceae, also at 9%; Polygonaceae, at 8%; Salicaceae, at 8%; Berberidaceae, at 6%; Fabaceae, at 6%; Poaceae, at 5%; and Grossulariaceae, also at 5%.

#### 3.2.2. Rust Fungi Geographical Components of the Sanjiangyuan Region

**Figure 2:**
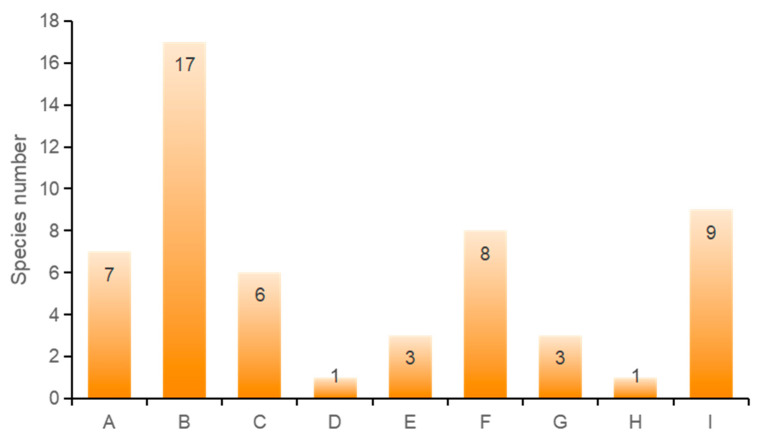
Analysis of geographic components of rust fungi in Sanjiangyuan. (A: Worldwide; B: Widely distributed in the northern temperate zone; C: The eurasian temperate zone is widespread; D: The northern hemisphere is cold and temperate; E: Central European component; F: East Asian elements; G: Central Asian component; H: Central and south Asian components; I: Chinese endemic).

The floristic geographical features primarily adhered to Wu Zhengyi’s plant floristic division principles [31]. The rust fungi of the forest plants in the Sanjiangyuan region have been preliminarily divided into nine geographical components. Cosmopolitan species account for 12.1%, North Temperate widespread species represent 30.9%, Eurasian Temperate widespread species comprise 10.9%, species widespread in both the cold and temperate zones of the Northern Hemisphere make up 1.8%, Central European components constitute 5.5%, East Asian components 14.5%, Central Asian components 5.5%, South Central Asian components 1.8%, and species endemic to China 16.4% (Figure 2).

#### 3.2.3. Comparative Analysis of Rust Fungi in the Sanjiangyuan Forests and Adjacent Regions

To ascertain the geographical composition of the rust fungi flora in the main forested areas of the Sanjiangyuan region, a comparison was made with rust fungi lists from neighboring areas (Table 3). The floristic elements of the rust fungi in the major forested areas of Sanjiangyuan show a higher similarity with those of Inner Mongolia, Gansu, and Tibet, with similarity coefficients of 49.6, 45.9, and 41.6, respectively. There is a moderate resemblance to the rust fungi flora of the Qinling Mountains, the Altai region in Xinjiang, and Jilin, with similarity coefficients of 38.2, 24.6, and 13.3, respectively. The disparity between the rust fungi flora of Hainan and the Sanjiangyuan region is substantial, with a coefficient of only 2.

### 3.3. Analysis of Life Forms of Rust Fungus Host Plants in the Sanjiangyuan

For the main forest regions, following the classification method of the “Flora of China”, the rust fungus host plants in the main forest regions of Sanjiangyuan are broadly categorized into three life forms: trees, shrubs, and herbaceous plants. The herbaceous plants dominate, comprising 17 families, 32 genera, and 44 species, accounting for 56.41% of the total species count; shrubs consist of 6 families, 11 genera, and 24 species, representing 30.77% of the total; trees include 4 families, 5 genera, and 8 species, making up 12.82%, as shown in Table 3, Table 4 and Table 5. It has been observed that the rust fungi parasitizing tree vegetation belong to three families, three genera, and six species, with the most diverse being the genus Melampsora, which primarily infects plants of the Salicaceae family. Those infecting shrub vegetation comprise 4 families, 5 genera, and 14 species, with the genus Gymnosporangium being the most diverse, affecting plants of the Rosaceae family. The species diversity of rust fungi parasitizing herbaceous vegetation is the highest, with 6 families, 8 genera, and 40 species, predominantly from the genus Puccinia, which afflicts plants of the Poaceae, Asteraceae, Gentianaceae, Polygonaceae, Ranunculaceae, Urticaceae, Rubiaceae, Onagraceae, Apiaceae, and Lamiaceae families.

### 3.4. Analysis of Rust Fungi in Different Regions of the Sanjiangyuan Area

**Figure 3:**
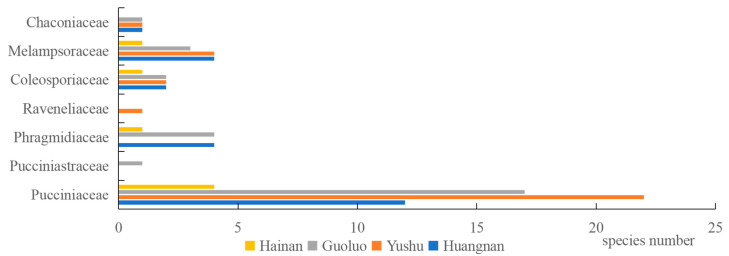
Spatial distribution of rust fungi in the main forest areas in Sanjiangyuan.

**Figure 4:**
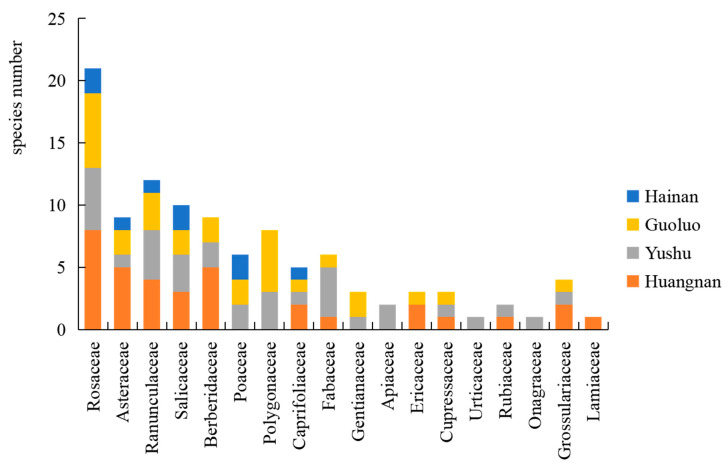
Spatial distribution of rust host plants in the main forest areas of Sanjiangyuan.

An investigation and analysis of rust fungi were conducted within four regions of the Sanjiangyuan area, revealing 7 families, 12 genera, and 56 species in total. Specifically, Golog Prefecture harbored 7 families, 9 genera, and 28 species; Yushu Prefecture was home to 6 families, 8 genera, and 31 species; Huangnan Prefecture contained 6 families, 9 genera, and 26 species; and Hainan Prefecture had 4 families, 5 genera, and 10 species. The families Pucciniaceae, Melampsoraceae, and Coleosporiaceae were common to rust fungi across all four regions (Figure 3), while the host plant families Rosaceae, Asteraceae, Ranunculaceae, Salicaceae, and Caprifoliaceae were shared among the regions (Figure 4).

#### 3.4.1. Rust Fungus Species Diversity

Upon collation and computation of the data, it was revealed that Yushu Prefecture boasts the richest diversity of rust fungus species, with a diversity index of 3.10. Within the four regions of the Sanjiangyuan area, the diversity indices of rust fungi, in descending order, are as follows: Yushu Prefecture, Guoluo Prefecture, Huangnan Prefecture, and Hainan Prefecture (Table 5).

#### 3.4.2. Rust Fungus Similarity Assessment across Different Regions

As analyzed in Table 6, the similarity coefficients for rust fungus species between Huangnan Prefecture and Guoluo Prefecture, Yushu Prefecture, and Hainan Prefecture are 0.5185, 0.4561, and 0.3333, respectively; between Guoluo Prefecture and Yushu Prefecture, Hainan Prefecture are 0.6102, and 0.2632, respectively; and between Yushu Prefecture and Hainan Prefecture is 0.1951. It is thus evident that the similarity in rust fungus species is highest between Yushu Prefecture and Guoluo Prefecture, and lowest between Yushu Prefecture and Hainan Prefecture.

## 4. Conclusions and Discussion

Upon surveying the forest mycoflora within the primary forests of the Sanjiangyuan region, a total of 56 rust fungus species and varieties across 7 families and 12 genera were identified. This inventory includes 1 new record for China, 2 proposed new species, and spans 26 families, 48 genera, and 78 species of host plants, with 10 plants being newly recorded hosts for these rust fungi. The dominant rust fungi families in the Sanjiangyuan forests are Pucciniaceae and Coleosporiaceae, and the prevalent genera include *Puccinia*, *Melampsora*, and *Gymnosporangium*. The host plants are predominantly from the Rosaceae, Asteraceae, Ranunculaceae, Polygonaceae, Salicaceae, Berberidaceae, Fabaceae, Poaceae, and Grossulariaceae families.

The phytoflora of this region is chiefly of the North Temperate Zone, and the discovery of the primitive rust fungus Hyalopsora adianti-capilli-veneris, which parasitizes on ferns, underscores the antiquity of the local flora. The geographic distribution of rust fungi in the Sanjiangyuan primary forests primarily consists of widespread North Temperate species, representing 17 species (30.9%). Followed by nine endemic Chinese species (16.4%), including *Gymnosporangium Huanglongense* and *Gymnosporangium pleoporum*, exclusive to the Sanjiangyuan region. There are eight East Asian species (14.5%), seven cosmopolitan species (12.7%), seix Eurasian Temperate widespread species (10.9%), and three each of the Central European and Central Asian components (5.5%, respectively). There are one species each (1.8%) from the North Hemisphere cold and temperate zones and the South and Central Asia components.

The Sanjiangyuan rust fungus flora shows high similarity coefficients with Inner Mongolia, Gansu, and Tibet, at 49.6, 45.9, and 41.6, respectively. This is likely due to geographical proximity and similar species composition, richness, and abundance. The similarity coefficient with the Qinling Mountains is 38.2, which may be attributed to its location between North and Southwest China, serving as a climatic divide, and its complex mountainous terrain with rich precipitation that supports a diverse ecosystem, hence a high diversity of fungi. The high altitude and harsh climatic conditions of the Sanjiangyuan region, characterized by plateaus, mountains, an arid climate, and low precipitation, result in relatively limited biodiversity. The similarity coefficient with Altai, Xinjiang is 24.6, possibly due to the diverse climate ranging from arid desert to temperate mountainous weather with clear seasonal changes and varied precipitation. The typical plateau climate of the Sanjiangyuan region has a significant impact on the survival and distribution of biota due to its cold climate, thin oxygen, low precipitation, and high evaporation. The dissimilarities in fungal diversity between the two regions are attributed to their different climatic conditions. With Jilin, the similarity coefficient is 13.3, likely because Jilin has a rich variety of ecosystems, including forests, wetlands, and grasslands, which support a wide range of plants, animals, and microorganisms. In contrast, the Sanjiangyuan region predominantly features plateau meadows, wetlands, and glaciers, hosting many endemic species but overall having less biodiversity than Jilin Province due to the severe climate. The rust fungi flora of Hainan shows a stark contrast with a similarity coefficient of only 2, as Hainan has a tropical monsoon climate with year-round warmth and moisture, abundant rainfall conducive to tropical rainforests, and other tropical ecosystems. The humid environment is ideal for many fungi species, unlike the typically fewer fungi found in the stark plateau climate of the Sanjiangyuan region.

In the Sanjiangyuan primary forests, herbaceous plants dominate the host plants for rust fungi, with 17 families, 32 genera, and 44 species (56.41%); shrubs comprise 6 families, 11 genera, and 24 species (30.77%); and trees make up 4 families, 5 genera, and 8 species (12.82%). It has been observed that rust fungi parasitizing arboreal vegetation include three families and three genera with six species, with *Melampsora* affecting the most species, particularly the Salicaceae plants. The shrub layer hosts 4 families, 5 genera, and 14 species of rust fungi, predominantly from *Gymnosporangium*, impacting the Rosaceae plants. The herbaceous layer carries the highest number of rust fungus species, with 6 families, 8 genera, and 40 species, with *Puccinia* being the most diverse, affecting Poaceae, Asteraceae, Gentianaceae, Polygonaceae, Ranunculaceae, Urticaceae, Rubiaceae, Onagraceae, Apiaceae, and Lamiaceae plants.

Diversity surveys of forest plant rust fungi across four distinct regions within the Sanjiangyuan revealed that biodiversity indices decreased in the order of Yushu Prefecture, Golog Prefecture, Huangnan Prefecture, and Hainan Prefecture. This pattern is attributed to the high coverage of pristine forests in Yushu Prefecture, offering complex and undisturbed natural habitats that provide rich conditions and biomass resources for a variety of fungi. The primeval forests of Yushu Prefecture, connected to the Hengduan Mountains in Tibet, furnish a diverse range of conditions and isolated environments for the distribution and evolution of species, favoring the maintenance of biodiversity and the emergence of endemic species. The highest similarity in rust fungi between Yushu and Golog Prefectures may be due to their shared primitive forests and similar altitudes, likely harboring comparable habitat types.

## Figures and Tables

**Table 1:** Rust Fungus Catalogue of the Major Forest Regions in the Sanjiangyuan Area.

Department	Genus	Species
Coleosporiaceae	*Chrysomyxa*	*Chrysomyxa woroninii* *
	*Coleosporium*	*Coleosporium pedicularis*
Chaconiaceae	*Ochropsora*	*Ochropsora ariae*
Melampsoraceae	*Melampsora*	*Melampsorella caryophyllacearum*, *Melampsora euphorbiae*, *Melampsora epitea*, *Melampsora kusanoi* *, *Melampsora larici-populina*, *Melampsora salicis*-*albae*, *Melampsora stellerae*
Raveneliaceae	*Nyssopsora*	*Nyssopsora asiatica* *
Phragmidiaceae	*Phragmidium*	*Phragmidium andersoni*, *Phragmidium potentillae*, *Phragmidium rubi*-*idaei*, *Phragmidium tuberculatum*
Pucciniaceae	*Gymnosporangium*	*Gymnosporangium annulatum, Gymnosporangium cornutum*, *Gymnosporangium confusum*, *Gymnosporangium huanglongense*, *Gymnosporangium pleoporum*, *Gymnosporangium yamadae*
	*Miyagia*	*Miyagia anaphalidis* *
	*Puccinia*	*Puccinia atragenes, Puccinia bistortae, Puccinia caricis*, *Puccinia circaeae, Puccinia calumnata*, *Puccinia coronata* var. coronate *, Puccinia chaerophylli *, *Puccinia dioicae*, *Puccinia festucae*, *Puccinia gentianae*, Puccinia graminis *, *Puccinia haleniae*, *Puccinia helianthi*, *Puccinia heraclei-nepalensis*, *Puccinia magnusiana*, *Puccinia polygoni-cyanandri*, *Puccinia recondita*, *Puccinia rhei-palmati*, *Puccinia rubiae-tataricae*, *Puccinia ribis* *, *Puccinia rupestris*, *Puccinia sorghi*, *Puccinia striiformis*, *Puccinia stipina*, *Puccinia* sp. (host plant: *Ligularia przewalskii*) **, *Puccinia* sp. (host plant: *Rheum pumilum*) **, *Puccinia vivipari*, Puccinia vomica
	*Uromyces*	*Uromyces hedysari-obscuri*, *Uromyces lapponicus*, *Uromyces lycoctoni*
Pucciniastraceae	*Hyalopsora*	*Hyalopsora adianti*-*capilli*-*veneris* ***
_	*Uredo*	*Uredo rhododendri*-*capitati*

* is a new host record, ** is a new species, *** is a new record in China.

**Table 2:** Composition of Rust Fungi families, genera, and species in Sanjiangyuan.

Department	Number of Genus	Percentage of Total Genera (%)	Number of Species	Percentage of Total Species (%)
Chaconiaceae	1	9.09	1	1.82
Coleosporiaceae	2	18.18	2	3.64
Melampsoraceae	1	9.09	7	12.73
Phragmidiaceae	1	9.09	4	7.27
Pucciniaceae	4	36.36	39	70.91
Pucciniastraceae	1	9.09	1	1.82
Raveneliaceae	1	9.09	1	1.82
total	11	100	55	100

**Table 3:** Comparison of rust flora in the main forest areas of Sanjiangyuan with adjacent areas.

Species	Sanjiangyuan	Inner Mongolia	Kansu	Tibet	Qinling	Xinjiang Altay	Jilin	Hainan
*Chrysomyxa woroninii*	+	-	-	-	-	-	-	-
*Coleosporium pedicularis*	+	+	-	+	+	-	-	-
*Gymnosporangium annulatum*	+	-	+	-	-	-	-	-
*G. cornutum*	+	+	-	-	-	+	-	-
*G. confusum*	+	-	-	+	+	+	-	-
*G. Huanglongense*	+	-	-	-	-	-	-	-
*G. pleoporum*	+	-	-	-	-	-	-	-
*G. yamadae*	+	+	+	-	+	-	+	-
*Hyalopsora adianti-capilli-veneris*	+	-	-	-	-	-	-	-
*Melampsorella caryophyllacearum*	+	-	+	-	-	+	-	-
*M. euphorbiae*	+	+	+	+	+	+	+	-
*M. epitea*	+	+	+	-	+	-	-	-
*M. kusanoi*	+	+	+	-	+	-	-	-
*M. larici-populina*	+	+	+	-	+	+	+	-
*M. salicis-albae*	+	+	+	-	-	-	-	-
*M. stellerae*	+	+	+	+	+	-	-	-
*Miyagia anaphalidis*	+	-	+	+	+	-	-	-
*Nyssopsora asiatica*	+	-	+	-	-	-	-	-
*Ochropsora ariae*	+	-	-	-	-	+	-	-
*Phragmidium andersoni*	+	+	+	+	-	+	-	-
*P. potentillae*	+	+	+	+	+	+	+	-
*P. rubi-idaei*	+	+	+	-	+	-	-	-
*P. tuberculatum*	+	-	+	+	-	-	-	-
*Puccinia atragenes*	+	+	-	+	-	-	-	-
*P. bistortae*	+	+	+	+	+	-	-	-
*P. calumnata*	+	+	-	-	-	-	-	-
*P. caricis*	+	+	+	+	+	-	+	-
*P. chaerophylli*	+	-	-	-	-	-	-	-
*P. circaeae*	+	-	+	+	-	-	+	-
*P. cnici-oleracei*	+	+	+	+	+	+	+	-
*P. coronata* var. *coronata*	+	+	-	+	+	-	-	+
*P. dioicae*	+	+	-	-	-	+	+	-
*P. festucae*	+	+	+	-	-	+	-	-
*P. gentianae*	+	+	+	+	-	+	-	-
*P. graminis*	+	+	+	+	+	+	+	-
*P. haleniae*	+	+	+	+	+	-	-	-
*P. helianthi*	+	+	+	-	+	-	+	-
*P. heraclei-nepalensis*	+	-	-	+	-	-	-	-
*P. magnusiana*	+	+	-	-	+	-	+	-
*P. polygoni-cyanandri*	+	-	+	-	-	-	-	-
*P. ribis*	+	-	-	+	+	+	-	-
*P. recondita*	+	+	+	+	+	-	+	-
*P. rhei-palmati*	+	-	-	-	+	-	-	-
*P. rubiae-tataricae*	+	-	-	+	-	-	-	-
*P. rupestris*	+	+	-	-	-	-	+	-
*P. sorghi*	+	+	+	+	+	-	-	+
*P. striiformis*	+	+	+	+	+	+	-	-
*P. stipina*	+	+	-	-	-	-	-	-
*Puccinia* sp. (*Ligularia przewalskii*)	+	-	-	-	-	-	-	-
*Puccinia* sp. (*Rheum pumilum*)	+	-	-	-	-	-	-	-
*P. vomica*	+	-	+	+	+	-	-	-
*P. vivipari*	+	-	+	-	-	-	-	-
*Uromyces hedysari-obscuri*	+	+	-	+	+	-	-	-
*U. lapponicus*	+	-	+	+	+	+	-	-
*U. lycoctoni*	+	-	-	-	+	+	-	-
*Uredo rhododendri-capitati*	+	-	-	-	+	-	-	-

**Table 4:** Analysis of host plant life form.

Plant Life Form	Species (Counting)	Percentage of Total Species (%)
herb	44	56.41
shrubs	24	30.77
arbor	10	12.82
total	78	100

**Table 5:** Rust population diversity in four regions.

	S (Species Numbers)	N (Abundance)	H′ (Diversity Index)	H′max (The Largest Species Diversity Index)	E (Uniformity)
Guoluo	28	52	3.08	3.95	0.7787
Hainan	10	25	2.28	3.22	0.7091
Huangnan	26	73	3.05	4.29	0.7117
Yushu	31	83	3.10	4.42	0.7024

**Table 6:** Scale factor.

	Huangnan	Guoluo	Yushu	Hainan
Huangnan	—	0.5185	0.4561	0.3333
Guoluo	0.5185	—	0.6102	0.2632
Yushu	0.4561	0.6102	—	0.1951
Hainan	0.3333	0.2632	0.1951	—

— Indicates that they cannot be compared.

## Data Availability

Data are contained within the article.

## Appendix A. Number of Rust Fungus Specimens Collected

**Order**	**Rust Fungi Species Name**	**Host Plants**	**Collection Sample Site**	**The Gatherer**	**Collection Number**
1	*Chrysomyxa woroninii*	*Rhododendron thymifolium*	Maixiu Forest Farm (101°56′08″ E, 35°54′35″ N)	Qi Xu; Hailan LI	QHU2022004, QHU2021135
2	*Coleosporium pedicularis*	*Pedicularis croizatiana*	Jiangxi Forest Farm (96°45′19″ E, 32°27′09″ N)	Qi Xu; Qinen He	QHU2022203, QHU2022219
		*Pedicularis croizatiana*	Lebagou Forest Farm (97°21′53″ E, 32°17′56″ N)	Qi Xu	QHU2022237
		*Pedicularis croizatiana*	Dongzhong Forest Farm (97°27′20″ E, 32°40′22”)	Qi Xu; Yuying Li	QHU2023065
		*Pedicularis croizatiana*	Yangyu Forest Farm (100°16′13″ E, 34°27′15″ N)	Qi Xu; Hailan LI	QHU2021007
3	*Gymnosporangium annulatum*	*Cotoneaster* sp.	Maixiu Forest Farm (101°56′08″ E, 35°54′35″ N)	Qi Xu; Hezhua Xiji	QHU2022134, QHU2021138, QHU2021129, QHU2021137, QHU2021131, QHU2021128
4	*G. cornutum*	*Sorbus koehneana*	Jiangxi Forest Farm (96°15′28″ E, 32°27′17″ N)	Qi Xu; Qinen He	QHU2022223
		*Sorbus koehneana*	Dongzhong Forest Farm (97°27′20″ E, 32°40′24″ N)	Qi Xu; Zihan Tan	QHU2023060
		*Sorbus koehneana*	Makehe Forest Farm (100°52′09″ E, 32°49′28″ N)	Qi Xu; Zihan Tan	QHU2023085
5	*G. confusum*	*Cotoneaster adpressus*	Jiangxi Forest Farm (96°15′28″ E, 32°27′18″ N)	Qi Xu; Qinen He	QHU2022217, QHU2022254, QHU2022255, QHU2021120
		*Cotoneaster multiflorus*	Jiangxi Forest Farm (96°15′28″ E, 32°27′35″ N)	Qi Xu; Qinen He	QHU2022244, QHU2022256, QHU2022257, QHU2021121, QHU2021122
6	*G. Huanglongense*	*Juniperus przewalskii*	Makehe Forest Farm (100°58′08″ E, 34°31′07″ N)	Qinen He	QHU2023023
		*Juniperus przewalskii*	Maixiu Forest Farm (101°16′59″ E, 35°28′42″ N)	Qi Xu; Qinen He	QHU2023024
7	*G. pleoporum*	*Cotoneaster acutifolius*	Jiangxi Forest Farm (96°15′33″ E, 32°27′30″ N)	Qinen He; Taijun Fang	QHU2022215
		*Cotoneaster acutifolius*	Maixiu Forest Farm (101°16′59″ E, 35°35′27″ N)	Fengying He; Hailan LI	QHU2022145, QHU2022162
		*Cotoneaster acutifolius*	Maixiu Forest Farm (101°09′41″ E, 35°27′56″ N)	Qi Xu; Qinen He	QHU2022014
		*Juniperus przewalskii*	Lebagou Forest Farm (97°21′56″ E, 32°12′07″ N)	Qinen He; Hailan LI	QHU2023025, QHU2022252, QHU2022253, QHU2021124, QHU2021118, QHU2021126
8	*G. yamadae*	*Pyrus* sp.	Maixiu Forest Farm (101°56′08″ E, 35°54′35″ N)	Qi Xu; Qinen He	QHU2022244, QHU2021123, QHU2021117, QHU2021125, QHU2021119, QHU2021127
9	*Hyalopsora adianti-capilli-veneris*	*Adiantum capillus-veneris*	Makehe Forest Farm (101°49′27″ E, 32°46′05″ N)	Qi Xu	QHU2023078, QHU2021133, QHU2021134, QHU2021139
10	*Melampsorella caryophyllacearum*	*Stellaria media*	Maixiu Forest Farm (101°29′16″ E, 35°20′18″ N)	Qi Xu	QHU2022111, QHU2021140, QHU2021144
11	*M. euphorbiae*	*Euphorbia micractina*	Lebagou Forest Farm (97°21′46″ E, 32°12′27″ N)	Fengying He; Shengshan Gan	QHU2022231, QHU2021146
12	*M. epitea*	*Salix sinica*	Jiangxi Forest Farm (98°46′42″ E, 36°37′29″ N)	Qi Xu; Hailan LI	QHU2022201
		*Salix sinica*	Jiangxi Forest Farm (98°46′42″ E, 36°37′30″ N)	Hezhua Xiji; Shengshan Gan	QHU2022226
		*Salix sinica*	Lebagou Forest Farm (97°21′38″ E, 32°12′07″ N)	Qi Xu	QHU2022242, QHU2021021
		*Salix sinica*	Dongzhong Forest Farm (96°29′33″ E, 31°49′12″ N)	Qi Xu; Zihan Tan	QHU2023064
		*Salix paraplesia*	Maixiu Forest Farm (101°09′38″ E, 35°27′05″ N)	Qinen He; Taijun Fang	QHU2022013, QHU2021037
		*Salix paraplesia*	Xihe Forest Farm (101°24′10″ E, 36°01′57″ N)	Qi Xu; Xiaoning Mao	QHU2023108
		*Salix oritrepha*	Lebagou Forestry Farm (97°20′16″ E, 32°13′02″ N)	Fengying He	QHU2022243
		*Salix oritrepha*	Yangyu Forest Farm (100°16′13″ E, 34°26′35″ N)	Qi Xu; Qinen He	QHU2021005, QHU2021016, QHU2021020
		*Salix oritrepha*	Yangyu Forest Farm (100°16′13″ E, 34°26′35″ N)	Qi Xu; Zihan Tan	QHU2023112, QHU2023113
		*Salix oritrepha*	Makehe Forest Farm (101°49′27″ E, 32°46′05″ N)	Qi Xu; Zihan Tan	QHU2023087
13	*M. kusanoi*	*Hypericum przewalskii*	Maixiu Forest Farm (101°22′08″ E, 35°25′17″ N)	Haixia Mu	QHU2022123, QHU2022194
14	*M. laricis-populina*	*Populus cathayana*	Maixiu Forest Farm (101°19′28″ E, 35°23′57″ N)	Qi Xu	QHU2022141, QHU2021038
		*Populus cathayana*	Jiangla Forest Farm (101°30′22″ E, 35°52′33″ N)	Qi Xu; Xiaoning Mao	QHU2023100, QHU2023107
15	*M. salicis-albae*	*Salix matsudana*	Maixiu Forest Farm (101°29′18″ E, 35°37′07″ N)	Qi Xu	QHU2022200, QHU2021143, QHU2021141
16	*M. stellerae*	*Stellera chamaejasme*	Jiangxi Forest Farm (96°14′02″ E, 32°27′49″ N)	Qi Xu	QHU2022205
		*Stellera chamaejasme*	Dongzhong Forest Farm (97°27′21″ E, 32°40′20″ N)	Qi Xu; Yuying Li	QHU2023062
		*Stellera chamaejasme*	Makehe Forest Farm (100°52′04″ E, 32°49′19″ N)	Qi Xu; Qinen He	QHU2021014
17	*Miyagia anaphalidis*	*Anaphalis lactea*	Maixiu Forest Farm (101°27′59″ E, 35°35′42″ N)	Qi Xu; Hailan LI	QHU2022136, QHU2022164
		*Anaphalis flavescens*	Maixiu Forest Farm (101°17′28″ E, 35°35′05″ N)	Qi Xu; Shengshan Gan	QHU2022158, QHU2021118, QHU2021119, QHU2021120, QHU2021121, QHU2021112
18	*Nyssopsora asiatica*	*Eleutherococcus wilsonii*	Jiangxi Forest Farm (96°09′47″ E, 32°29′38″ N)	Qi Xu; Qinen He	QHU2022221, QHU2021132
19	*Ochropsora ariae*	*Anemone rivularis* var. *flore-minore*	Maixiu Forest Farm (101°54′34″ E, 35°16′19″ N)	Qi Xu; Shengshan Gan	QHU2022146
		*Anemone rivularis* var. *flore-minore*	Jiangxi Forest Farm (96°55′45″ E, 32°15′36″ N)	Qi Xu; Qinen He	QHU2022206
		*Anemone rivularis* var. *flore-minore*	Dongzhong Forest Farm (97°27′19″ E, 32°40′19″ N)	Qi Xu; Yuying Li	QHU2023055
		*Anemone rivularis* var. *flore-minore*	Makehe Forest Farm (100°52′04″ E, 32°49′19″ N)	Qi Xu; Qinen He	QHU2021032, QHU2021078
20	*Phragmidium andersoni*	*Dasiphora fruticosa*	Maixiu Forest Farm (101°55′18″ E, 35°53′35″ N)	Qinen He; Hezhua Xiji	QHU2023105, QHU2021136, QHU2021130
21	*P. potentillae*	*Potentilla saundersiana*	Maixiu Forest Farm (101°56′18″ E, 35°25′51″ N)	Qi Xu; Qinen He	QHU2022019
		*Potentilla saundersiana*	Makehe Forest Farm (100°52′12″ E, 32°49′33″ N)	Qi Xu; Qinen He	QHU2021054
		*Potentilla multifida*	Maixiu Forest Farm (101°09′27″ E, 35°26′53″ N)	Taijun Fang; Hailan LI	QHU2022037
		*Potentilla multifida*	Jiangxi Forest Farm (96°14′47″ E, 32°27′15″ N)	Qi Xu	QHU2022202, QHU2022229
22	*P. rubi-idaei*	*Rubus sachalinensis*	Maixiu Forest Farm (101°26′16″ E, 35°26′52″ N)	Qinen He; Taijun Fang	QHU2022151
		*Rubus sachalinensis*	Makehe Forest Farm (100°52′09″ E, 32°49′30″ N)	Qi Xu; Qinen He	QHU2023084
		*Rubus sachalinensis*	Xihe Forest Farm (101°34′34″ E, 36°15′26″ N)	Qi Xu; Xiaoning Mao	QHU2023102
23	*P. tuberculatum*	*Rosa omeiensis*	Maixiu Forest Farm (101°22′46″ E, 35°24′46″ N)	Qinen He; Taijun Fang	QHU2022012, QHU2022036
		*Rosa omeiensis*	Maixiu Forest Farm (101°43′44″ E, 36°29′41″ N)	Qi Xu; Shengshan Gan	QHU2022144, QHU2022170
		*Rosa omeiensis*	Makehe Forest Farm (100°52′15″ E, 32°49′29″ N)	Qi Xu; Qinen He	QHU2021026
		*Rosa giraldii*	Maixiu Forest Farm (101°03′13″ E, 35°24′06″ N)	Qi Xu	QHU2022024, QHU2022001, QHU2022066, QHU2022094
		*Rosa* sp.	Maixiu Forest Farm (101°52′20″ E, 35°21′14″ N)	Qi Xu; Hezhua Xiji	QHU2022157
		*Rosa* sp.	Yangyu Forest Farm (100°16′11″ E, 34°27′21″ N)	Qi Xu; Qinen He	QHU2021010
		*Rosa* sp.	Makehe Forest Farm (100°49′28″ E, 32°46′06″ N)	Qi Xu; Qinen He	QHU2023079
		*Rosa* sp.	Makehe Forest Farm (100°49′28″ E, 32°46′05″ N)	Qi Xu; Liming Zhang	QHU2023081
		*Rosa* sp.	Makehe Forest Farm (100°57′32″ E, 32°40′55″ N)	Qi Xu; Zihan Tan	QHU2023089
		*Rosa* sp.	Xihe Forest Farm (101°34′32″ E, 36°15′30″ N)	Qi Xu; Xiaoning Mao	QHU2023099
24	*Puccinia atragenes*	*Clematis rehderiana*	Jiangxi Forest Farm (96°54′33″ E, 32°16′29″ N)	Haixia Mu; Hezhua Xiji	QHU2022208, QHU2021090, QHU2021087, QHU2021091
25	*P. bistortae*	*Bistorta vivipara*	Maixiu Forest Farm (101°50′31″ E, 35°26′25″ N)	Qi Xu; Shengshan Gan	QHU2023070
		*Bistorta vivipara*	Dongzhong Forest Farm (96°29′34″ E, 31°49′12″ N)	Qi Xu; Yuying Li	QHU2023046
		*Bistorta vivipara*	Makehe Forest Farm (100°44′28″ E, 32°56′03″ N)	Qi Xu; Zihan Tan	QHU2022238
		*Bistorta vivipara*	Yangyu Forest Farm (100°33′36″ E, 34°32′57″ N)	Qi Xu; Yuying Li	QHU2022207
26	*P. calumnata*	*Koenigia divaricata*	Lebagou Forest Farm (97°16′26″ E, 32°55′21″ N)	Taijun Fang	QHU2022232
		*Koenigia divaricata*	Dongzhong Forest Farm (97°27′19″ E, 32°40′24″ N)	Qi Xu; Yuying Li	QHU2023063
27	*P. caricina*	*Urtica triangularis*	Lebagou Forest Farm (97°12′50″ E, 32°54′44″ N)	Qi Xu	QHU2022007, QHU2022067, QHU2022241, QHU2021099
		*Ribes himalense*	Maixiu Forest Farm (101°54′35″ E, 35°16′19″ N)	Qi Xu; Shengshan Gan	QHU2022010, QHU2022152
		*Ribes stenocarpum*	Maixiu Forest Farm (101°54′36″ E, 35°16′19″ N)	Qi Xu; Wenbo Leng	QHU2022011
		*Ribes stenocarpum*	Maixiu Forest Farm (101°52′39″ E, 35°15′50″ N)	Haixia Mu; Hezhua Xiji	QHU2022153
		*Ribes* sp.	Makehe Forest Farm (100°49′26″ E, 32°45′11″ N)	Qi Xu; Qinen He	QHU2022010, QHU2022135, QHU2022152, QHU2021004
28	*P. chaerophylli*	*Anthriscus sylvestris*	Jiangxi Forest Farm (96°27′11″ E, 32°15′18″ N)	Qi Xu	QHU2022228, QHU2021113, QHU2021114, QHU2021107, QHU2021115, QHU2021109
29	*P. circaeae*	*Circaea alpina*	Jiangxi Forest Farm (96°27′08″ E, 32°15′28″ N)	Taijun Fang; Shengshan Gan	QHU2022218, QHU2022220
		*Circaea alpina*	Jiangxi Forest Farm (96°32′26″ E, 31°50′07″ N)	Qi Xu; Yuying Li	QHU2023117
30	*P. cnici-oleracei*	*Saussurea* sp.	Maixiu Forest Farm (101°52′11″ E, 35°24′17″ N)	Qi Xu	QHU2022060, QHU2022062, QHU2022106, QHU2022143
31	*P. coronata* var. *coronata*	*Clematis* sp.	Dongzhong Forest Farm (97°27′19″ E, 32°40′24″ N)	Qi Xu; Zihan Tan	QHU2022059, QHU2023068
32	*P. dioicae*	Asteraceae	Maixiu Forest Farm (101°52′13″ E, 35°14′59″ N)	Qi Xu; Fengying He	QHU2022022, QHU2022038, QHU2022040
33	*P. festucae*	*Lonicera* sp.	Maixiu Forest Farm (101°54′35″ E, 35°16′21″ N)	Qi Xu; Qinen He	QHU2022009, QHU2022023, QHU2022029, QHU2022032
		*Lonicera* sp.	Dongshan Forest Farm (101°36′57″ E, 36°13′50″ N)	Qi Xu; Xiaoning Mao	QHU2023011
		*Lonicera* sp.	Lebagou Forest Farm (97°16′31″ E, 32°55′22″ N)	Qi Xu; Qinen He	QHU2021025
		*Lonicera hispida*	Jiangxi Forest Farm (96°54′53″ E, 32°16′40″ N)	Qi Xu	QHU2022209
		*Lonicera hispida*	Lebagou Forest Farm (97°16′33″ E, 32°55′18″ N)	Qi Xu; Qinen He	QHU2022234, QHU2023020
		*Lonicera hispida*	Dongzhong Forest Farm (96°30′59″ E, 31°48′42″ N)	Qi Xu; Zihan Tan	QHU2023058
		*Lonicera hispida*	Makehe Forest Farm (100°52′03″ E, 32°49′30″ N)	Qi Xu; Zihan Tan	QHU2023018, QHU2022134
		*Lonicera tangutica*	Maixiu Forest Farm (101°54′36″ E, 35°16′09″ N)	Qi Xu; Fengying He	QHU2022009, QHU2022023
34	*P. gentianae*	*Gentiana straminea*	Lebagou Forest Farm (97°20′18″ E, 32°13′08″ N)	Qi Xu; Taijun Fang	QHU2022233, QHU2021102, QHU2021100, QHU2021104
35	*P. graminis*	*Berberis dasystachya*	Maixiu Forest Farm (101°54′31″ E, 35°16′21″ N)	Qi Xu; Qinen He	QHU2022008, QHU2022031, QHU2022070, QHU2022110, 2021097
		*Berberis poiretii*	Maixiu Forest Farm (101°54′36″ E, 35°15′20″ N)	Qi Xu	QHU2022026, QHU2022077, QHU2022188, QHU2022017
		*Berberis diaphana*	Maixiu Forest Farm (101°54′41″ E, 35°15′22″ N)	Qi Xu; Qinen He	QHU2022169
		*Berberis diaphana*	Jiangxi Forest Farm (96°54′36″ E, 32°17′38″ N)	Qi Xu	QHU2022230, QHU2022239
		*Berberis vulgaris*	Maixiu Forest Farm (101°54′41″ E, 35°15′26″ N)	Qi Xu; Wenbo Leng	QHU2022168
		*Berberis vulgaris*	Jiangxi Forest Farm (96°55′33″ E, 32°47′04″ N)	Qi Xu; Fengying He	QHU2022213
		*Berberis vulgaris*	Makehe Forest Farm (100°49′27″ E, 32°46′07″ N)	Qi Xu; Qinen He	QHU2021098, QHU2021101, QHU2021103
36	*P. haleniae*	*Halenia elliptica*	Jiangxi Forest Farm (96°54′ 55″ E, 32°16′39″ N)	Qi Xu	QHU2023123, QHU2021095
37	*P. helianthi*	*Helianthus annuus*	Jiangla Forest Farm (101°30′27″ E, 35°51′55″ N)	Qi Xu; Xiaoning Mao	QHU2023095, QHU2021094, QHU2021086
38	*P. heraclei-nepalensis*	*Heracleum candicans*	Dongzhong Forest Farm (96°32′31″ E, 31°50′12″ N)	Qinen He; Liming Zhang	QHU2023053
		*Heracleum candicans*	Lebagou Forest Farm (97°12′12″ E, 32°54′46″ N)	Qinen He; Taijun Fang	QHU2021019
39	*P. magnusiana*	*Phragmites australis*	Xihe Forest Farm (101°24′07″ E, 36°01′53″ N)	Qi Xu; Xiaoning Mao	QHU2023098, QHU2021083, QHU2021070
40	*P. polygoni-cyanandri*	*Koenigia cyanandra*	Makehe Forest Farm (100°52′07″ E, 32°49′31″ N)	Qi Xu; Qinen He	QHU2023082, QHU2023001, QHU2021080, QHU2021081
41	*P. ribis*	*Ribes glaciale*	Jiangxi Forest Farm (96°15′28″ E, 32°27′53″ N)	Qi Xu; Fengying He	QHU2022224, QHU2023116, QHU2021110, QHU2021111
42	*P. recondita*	*Aquilegia viridiflora*	Maixiu Forest Farm (101°52′17″ E, 35°21′06″ N)	Qi Xu; Shengshan Gan	QHU2022160, QHU2021085, QHU2021096, QHU2021092
		*Thalictrum alpinum*	Maixiu Forest Farm (101°52′22″ E, 35°21′13″ N)	Qi Xu	QHU2022199
		*Thalictrum alpinum*	Makehe Forest Farm (100°51′13″ E, 32°49′10″ N)	Qinen He; Taijun Fang	QHU2021051
		*Thalictrum aquilegiifolium var. sibiricum*	Maixiu Forest Farm (101°54′37″ E, 35°16′08″ N)	Qi Xu	QHU2022016, QHU2022167, QHU2022210
		*Thalictrum aquilegiifolium var. sibiricum*	Jiangxi Forest Farm (96°54′12″ E, 32°16′33″ N)	Qi Xu; Shengshan Gan	QHU2022225, QHU2023067, QHU2021027
		*Thalictrum aquilegiifolium var. sibiricum*	Dongzhong Forest Farm (96°32′27″ E, 31°50′07″ N)	Qi Xu; Qinen He	QHU2021082
		*Thalictrum aquilegiifolium var. sibiricum*	Makehe Forest Farm (100°51′33″ E, 32°49′19″ N)	Qi Xu; Qinen He	QHU2021060, QHU2021089, QHU2021070
		*Agropyron cristatum*	Jiangxi Forest Farm (96°54′19″ E, 32°16′59″ N)	Qi Xu; Fengying He	QHU2022222
		*Agropyron cristatum*	Lebagou Forest Farm (97°12′16″ E, 32°54′44″ N)	Haixia Mu; Hezhua Xiji	QHU2022240
		*Agropyron cristatum*	Dongzhong Forest Farm (96°32′25″ E, 31°50′06″ N)	Qi Xu; Yuying Li	QHU2023054
		*Agropyron cristatum*	Makehe Forest Farm (100°52′10″ E, 32°49′29″ N)	Qi Xu; Qinen He	QHU2022183, QHU2022212, QHU2023074
		*Agropyron cristatum*	Yangyu Forest Farm (100°16′12″ E, 34°27′19″ N)	Qi Xu; Qinen He	QHU2023021, QHU20210093
43	*P. rhei-palmati*	*Rheum tanguticum*	Makehe Forest Farm (100°52′10″ E, 32°49′27″ N)	Qi Xu; Liming Zhang	QHU2023086, QHU2021088, QHU2021084
44	*P. rubiae-tataricae*	*Rubia cordifolia*	Maixiu Forest Farm (101°50′29″ E, 35°26′31″ N)	Qi Xu; Qinen He	QHU2022112, QHU2022119
		*Rubia cordifolia*	Jiangxi Forest Farm (96°14′11″ E, 32°27′12″ N)	Qi Xu	QHU2022211
45	*P. rupestris*	*Saussurea* sp.	Maixiu Forest Farm (101°52′11″ E, 35°24′17″ N)	Qi Xu	QHU2022041, QHU2021170
46	*P. sorghi*	*Zea mays*	Jiangla Forest Farm (101°30′21″ E, 35°51′21″ N)	Qi Xu; Xiaoning Mao	QHU2023096, QHU2023097
47	*P. striiformis*	*Berberis circumserrata*	Maixiu Forest Farm (101°54′30″ E, 35°16′21″ N)	Qi Xu; Qinen He	QHU2022018, QHU2022028
		*Berberis circumserrata*	Makehe Forest Farm (100°49′27″ E, 32°46′16″ N)	Qinen He; Hailan LI	QHU2022097
		*Leymus secalinus*	Jiangxi Forest Farm (96°54′53″ E, 32°16′40″ N)	Qi Xu; Fengying He	QHU2022204, QHU2021023
		*Leymus secalinus*	Makehe Forest Farm (100°49′26″ E, 32°45′07″ N)	Qi Xu	QHU2021008
48	*P. stipina*	*Dracocephalum heterophyllum*	Maixiu Forest Farm (101°08′28″ E, 35°27′26″ N)	Qi Xu; Taijun Fang	QHU2022130, QHU202110105, QHU2021106, QHU2021112, QHU2021108
49	*Puccinia* sp.	*Ligularia przewalskii*	Makehe Forest Farm (100°57′33″ E, 32°40′54″ N)	Qi Xu; Qinen He	QHU2022139, QHU2023090, QHU2021116
50	*Puccinia* sp.	*Rheum pumilum*	Yangyu Forest Farm (100°33′37″ E, 34°32′56″ N)	Qi Xu; Hailan LI	QHU2023094, QHU2021055
51	*P. vomica*	*Saussurea epilobioides*	Maixiu Forest Farm (101°54′37″ E, 35°16′21″ N)	Qi Xu; Qinen He	QHU2022143, QHU2022148, QHU2022155, QHU2022158
		*Saussurea epilobioides*	Makehe Forest Farm (100°52′08″ E, 32°49′29″ N)	Qi Xu; Qinen He	QHU2022159, QHU2022164, QHU2023077
52	*P. vivipari*	*Bistorta vivipara*	Lebagou Forest Farm (97°23′14″ E, 32°22′45″ N)	Qi Xu; Shengshan Gan	QHU2021029
		*Bistorta vivipara*	Jiangxi Forest Farm (96°14′02″ E, 32°27′18″ N)	Qi Xu; Fengying He	QHU2023091
		*Bistorta vivipara*	Makehe Forest Farm (100°49′31″ E, 32°45′16″ N)	Qi Xu; Qinen He	QHU2023071
53	*Uromyces hedysari-obscuri*	*Hedysarum sikkimense*	Lebagou Forest Farm (97°22′46″ E, 32°13′42″ N)	Qi Xu; Fengying He	QHU2022235, QHU2022236
		*Hedysarum polybotrys* var. *alaschanicum*	Yangyu Forest Farm (100°33′37″ E, 34°32′52″ N)	Qi Xu; Qinen He	QHU2021003
		*Hedysarum polybotrys* var. *alaschanicum*	Lebagou Forest Farm (97°22′45″ E, 32°13′39″ N)	Qi Xu; Qinen He	QHU2023120
		*Astragalus* sp.	Dongzhong Forest Farm (97°27′19″ E, 32°40′24″ N)	Qi Xu; Yuying Li	QHU2023057
		*Astragalus* sp.	Lebagou Forest Farm (97°22′36″ E, 32°13′47″ N)	Qi Xu; Qinen He	QHU2021024, QHU2021022
54	*U. lapponicus*	*Astragalus* sp.	Maixiu Forest Farm (101°26′19″ E, 35°26′52″ N)	Qi Xu; Qinen He	QHU2022150, QHU2022249, QHU2022250, QHU2021113, QHU2021114
		*Oxytropis* sp.	Maixiu Forest Farm (101°10′31″ E, 35°26′25″ N)	Shengshan Gan; Haixia Mu	QHU2022080, QHU2022251, QHU2021115, QHU2021117
55	*U. lycoctoni*	*Aconitum sinomontanum*	Jiangla Forest Farm (101°36′57″ E, 36°13′50″ N)	Qi Xu; Xiaoning Mao	QHU2023003, QHU2023104
56	*Uredo rhododendri-capitati*	*Rhododendron capitatum*	Maixiu Forest Farm (101°57′08″ E, 35°54′35″ N)	Qi Xu; Hailan LI	QHU2022003, QHU2021142, QHU2021145, QHU2021147
